# The Use of Solitaire AB Stents in Coil Embolization of Wide-Necked Cerebral Aneurysms

**DOI:** 10.1371/journal.pone.0139714

**Published:** 2015-10-01

**Authors:** Teng-Fei Li, Shao-Feng Shui, Xin-Wei Han, Lei Yan, Ji Ma, Dong Guo, Hong-Can Zhu, Shu-Kai Wang, Yuan-Hong He, Wen-Wu Chen, Li-Ping Wei, Ming-Ke Wang, Tai-Min Song

**Affiliations:** 1 Department of Interventional Radiology, the First Affiliated Hospital of Zhengzhou University, Zhengzhou, China; 2 Interventional Institute of Zhengzhou University, Zhengzhou, China; 3 Department of Neurology, The First Affiliated Hospital of Zhengzhou University, Zhengzhou, China; 4 Department of Neurosurgery, The First Affiliated Hospital of Zhengzhou University, Zhengzhou, China; 5 Department of Neurology, The Fifth Affiliated Hospital of Zhengzhou University, Zhengzhou, China; 6 Department of Neurology, The Affiliated First Hospital of Henan University, Kaifeng, China; 7 Department of Neurology, Luoyang Central Hospital, Luoyang, China; 8 Department of Neurology, Luohe First people’s Hospital, Luohe, China; University of Washington, UNITED STATES

## Abstract

**Background:**

The Solitaire AB stent is one of many assistant stents used for treating wide-necked cerebral aneurysm, and has been used since 2003. However, large sample studies on its safety and effectiveness are lacking. The objective of this study was to evaluate the effectiveness and safety of the Solitaire AB stent in the coil embolization of wide-necked cerebral aneurysms.

**Methods:**

Retrospective review of the clinical and image data of 116 patients with wide-necked cerebral aneurysms who had been enrolled at six interventional neuroradiology centers from February 2010 to February 2014 and had been treated by coil embolization; in total, 120 Solitaire AB stents were used. The degree of aneurysm occlusion was examined using digital subtraction angiography (DSA) immediately after the procedure and during follow-up, and was graded using the modified Raymond classification. We also observed complications to evaluate the safety and effectiveness of this therapy.

**Results:**

The 120 Solitaire AB stents (4 mm × 15 mm, four stents; 4 mm × 20 mm, 16 stents; 6 mm × 20 mm, 36 stents; 6 mm × 30 mm, 64 stents) were inserted to treat 120 wide-necked cerebral aneurysms. All stents were inserted successfully. DSA immediately post-surgery revealed 55 cases of complete occlusion, 59 cases of neck remnant, and six cases of aneurysm remnant. Perioperatively, there were four cases of hemorrhage and four cases of stent thrombosis. The follow-up spanned 3–37 months; of 92 patients examined by DSA at the 6-month follow up, 12 had disease recurrence.

**Conclusions:**

The Solitaire AB stent is effective with a good technical success rate and short-term effect for assisting coil embolization of wide-necked cerebral aneurysms.

## Introduction

Intravascular interventional embolization has become an important means of treating intracranial aneurysm; the International Subarachnoid Aneurysm Trial has confirmed its safety and effectiveness [1]. However, embolizing wide-necked aneurysms (neck width > 4 mm or dome:neck < 2) with only coils remains technically challenging. Stents can facilitate embolization surgery and promote healing of the aneurysm neck, thereby resolving the issue. The Solitaire AB stent (eV3, Irvine, CA) is one of many assistant stents, and has been used since 2003 [2]; it has a tubular laser-cut design with a closed-cell structure and retrievability before Guglielmi detachment; its use is widespread and it has good effectiveness [3–6]. However, there is a lack of large sample studies on its safety and effectiveness [3–5]. In this study, we retrospectively reviewed the clinical and image data of 116 patients with wide-necked cerebral aneurysms who had been enrolled at six interventional neuroradiology centers from February 2010 to February 2014 and treated by coil embolization with 120 Solitaire AB stents in total, and we evaluated safety and effectiveness of the stent.

## Methods

### Ethics Statement

Written informed consent was obtained from each patient for the publication of this paper and any accompanying images. The ethics committee of each center approved this study (the ethics committee of the First Affiliated Hospital of Zhengzhou University; the ethics committee of the Fifth Affiliated Hospital of Zhengzhou University; the ethics committee of the First Affiliated Hospital of Henan University; the ethics committee of Luoyang Central Hospital of Zhengzhou University; the ethics committee of the First Affiliated Hospital of Luohe Medical College) (S1 and S2 Figs, S1 Text). The procedures followed were in accordance with the Helsinki Declaration of 1975, as revised in 1983.

### Patients

Of the 116 patients in this study, 83 were male and 33 were female; the mean age was 55.9 ± 12.1 years. There were 72 unruptured (including four cases of two wide-necked aneurysms in one patient) and 48 ruptured aneurysms. The clinical severity of subarachnoid hemorrhage was assessed using the Hunt and Hess grade: grade I, 16 cases; grade II, 28 cases; grade III, four cases.

### Aneurysms

Of the 120 aneurysms, 82 were in the anterior circulation: four in the lacerum segment (C3) of the internal carotid artery (ICA), six in the intracavernous ICA, 13 in the supraclinoid ICA, 29 in the carotid-ophthalmic section, 23 in the intracavernous carotid, two in the ICA bifurcation, and five in the middle cerebral artery (MCA). There were 38 aneurysms in the posterior circulation: 11 in the vertebral artery, 14 in the basilar trunk, five in the top basilar artery, and eight in the posterior cerebral artery. The spatial relation between aneurysms and the parent artery and its branches was confirmed by digital subtraction angiography (DSA) and its three-dimensional reconstruction function (Table 1).

**Table 1:** Aneurysm measurement.

	Mean±SD(mm)
Dome width	4.6±1.6
Dome-to-neck ratio	0.7±0.2
Proximal parent artery	4.0±0.6
Distal parent artery	3.8±0.6

### Perioperative Management

Patients with unruptured aneurysms were given 100 mg/d aspirin and 75 mg/d clopidogrel for five days before intervention; patients with ruptured aneurysms were given 300 mg clopidogrel and 300 mg aspirin at least 2 h and not more than 3 h before intervention; patients in a coma were given the above medicines rectally after anesthesia. Full heparinization was achieved in the whole procedure; specifically, the first dose of heparin was 4000 U, which rendered the activated clotting time > 250 ms, or 2.5 times the normal value. Then, the dosage was halved per 1 h to a minimal dose of 1000 U/h, and the dose was maintained. Another 2000 U heparin was given after stent placement. After the procedure, vasospasm prevention and symptomatic and supportive treatment were administered, and patients took 75 mg/d clopidogrel for six weeks and used 100 mg/d aspirin in the long-term.

### Procedure

The size of the Solitaire AB stents was determined by the surgeon according to the vessel diameter and the length of the neck. The jailing technique was used for the insertion of 83 stents, i.e., following introduction with a micro-guide wire, the stent was inserted at the aneurysm neck, a microcatheter was placed in the aneurysm, and the coils were inserted through the catheter, but not detached. If the location of the stent was not good or the coils emerged in the parent artery, the stent was withdrawn with a Rebar catheter and its location was adjusted before it was re-released, pushing the coils back to the intracavity of the aneurysm. Then, coil embolization continued until the aneurysm had been completely embolized, following which the stent was eventually fully released. We treated 37 aneurysms using the trans-stent technique, i.e., stent placement was followed by microcatheter placement in the sac through the stent struts.

### Postoperative Evaluation and Follow-up

The degree of aneurysm occlusion was evaluated through DSA immediately after the procedure and at the 6-month follow-up, which was concluded by two senior professional physicians (Shao-feng Shui, 30 years’ experience; Xin-wei Han, 35 years’ experience), and scored using the modified Raymond classification [7]. Complete occlusion was defined as the absence of contrast agent in the aneurysm intracavity; a neck remnant was contrast agent at the aneurysm neck; aneurysm remnant referred to the portion of the aneurysm that was filled with contrast agent. Clinical outcome was assessed at discharge and then at six months using the modified Rankin Scale (mRS). Any death within 30 days after endovascular treatment was deemed treatment-related death.

## Results

### Technical and Anatomic Postoperative Results

**Fig 1:**
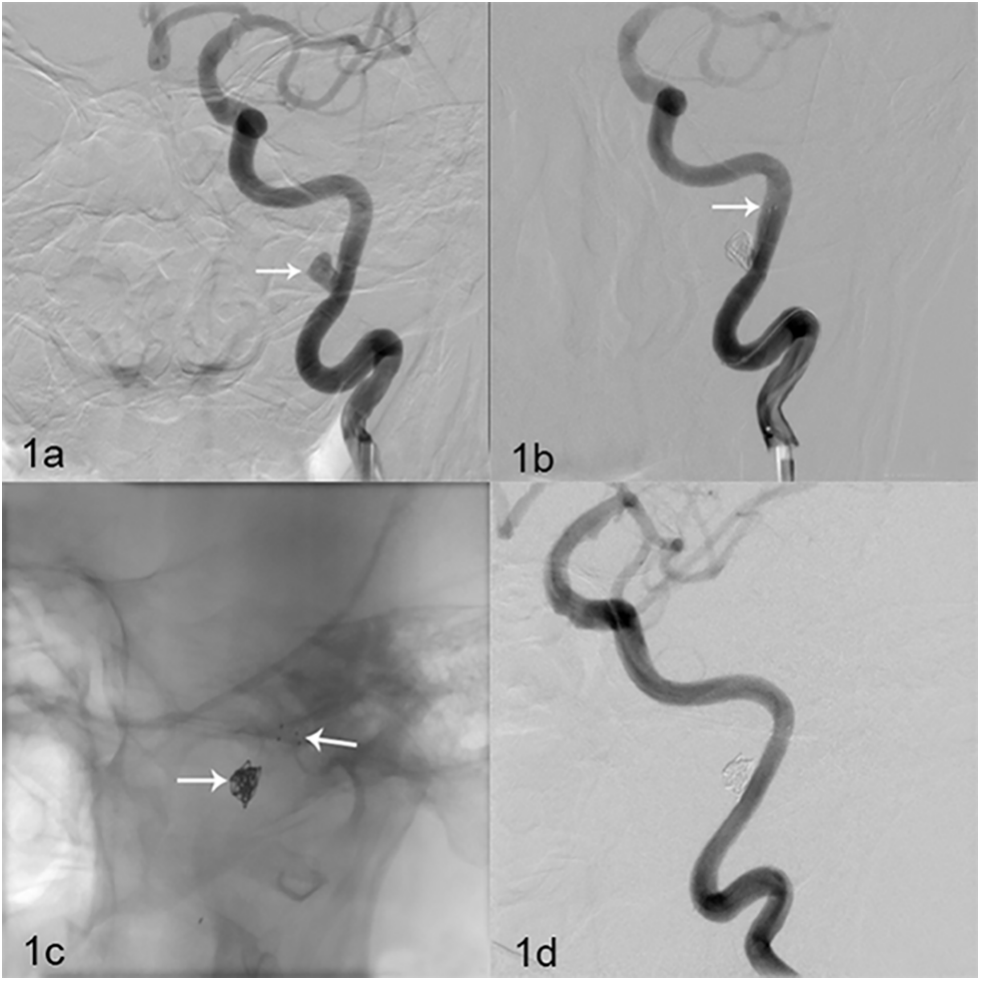
Solitaire AB stent (6 mm × 30 mm) assisting complete occlusion in one patient. **(a)** Figure depicts a 43-year-old woman with an unruptured aneurysm. DSA revealed a left C3 artery wide-necked aneurysm (6.5 mm × 4.2 mm) (white arrow). (b) Solitaire AB stent (6 mm × 30 mm) (white arrow) assisting embolization. DSA immediately after the procedure revealed complete occlusion of the aneurysm and parent artery patency. (c-d) DSA at the 6-month follow-up revealed no aneurysm development, and revealed parent artery patency.

The 116 patients with 120 wide-necked aneurysms received 120 Solitaire AB stents (four stents, 4 mm × 15 mm; 16 stents, 4 mm × 20 mm; 36 stents, 6 mm × 20 mm; 64 stents, 6 mm × 30 mm). All stents were inserted successfully: 97 were inserted once successfully, while 23 were released twice because the coils emerged in the parent artery. Stents were Guglielmi-detached once successfully in 94 cases, twice successfully in 17 cases, and thrice successfully in 10 cases, with detachment time ranging 41–272 s (average, 107 s). DSA immediately after the procedure showed that 55 cases had complete occlusion (45.8%, 55/120) (Fig 1A–1D), 59 cases had neck remnants (49.2%, 59/120), and six had aneurysm remnants (5.0%, 6/120).

### Perioperative Complications

**Fig 2:**
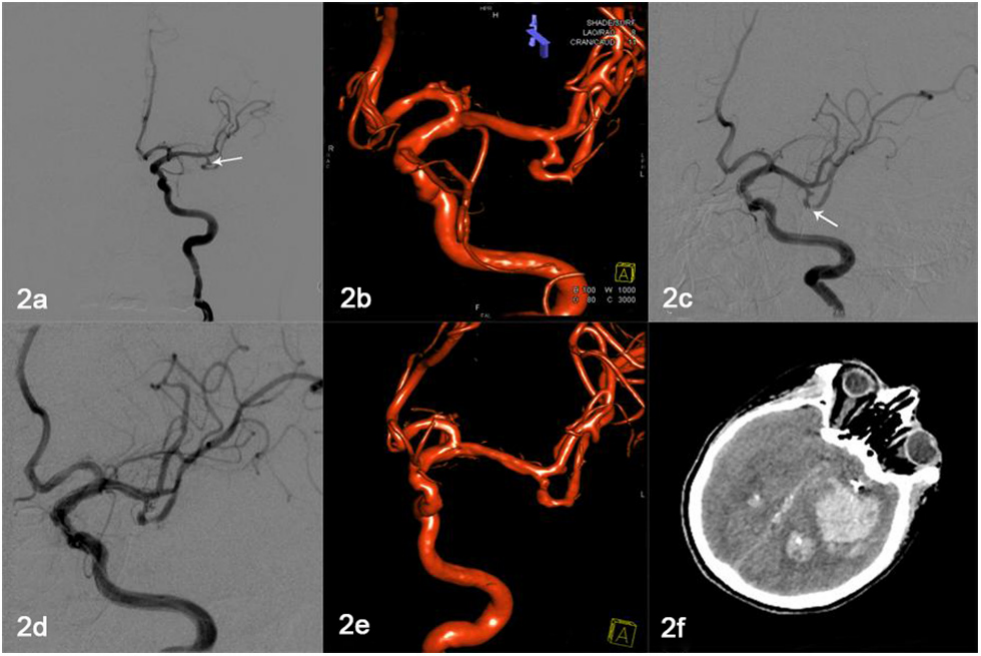
Hemorrhage after stenting in one patient. **(a–b)** Figure depicts a 65-year-old man with a left MCA ruptured wide-necked aneurysm (2.5 mm × 3.2 mm) (white arrow). **(c–e)** Solitaire AB stent (4 mm × 20 mm) (white arrow) assisting complete occlusion; there was parent artery patency. (f) CT revealing hemorrhage in the left MCA area 5 h after surgery.

Eight patients had perioperative complications (6.9%, 8/116). Among them, four were hemorrhagic: there were three cases of perioperative aneurysm rupture, which were resolved by the insertion of more coils; computed tomography (CT) scan immediately after the procedure revealed a small amount of subarachnoid hemorrhage, and the patients only suffered a mild headache. Aneurysm rerupture was detected in the fourth patient, who died from herniation after surgery because his family refused craniotomy. This patient had vomited and had gone into a coma 5 h after stenting; CT revealed intracerebral hemorrhage around the stent, and the patient died 2 h after that because of cerebral hernia (Fig 2A–2F).

**Fig 3:**
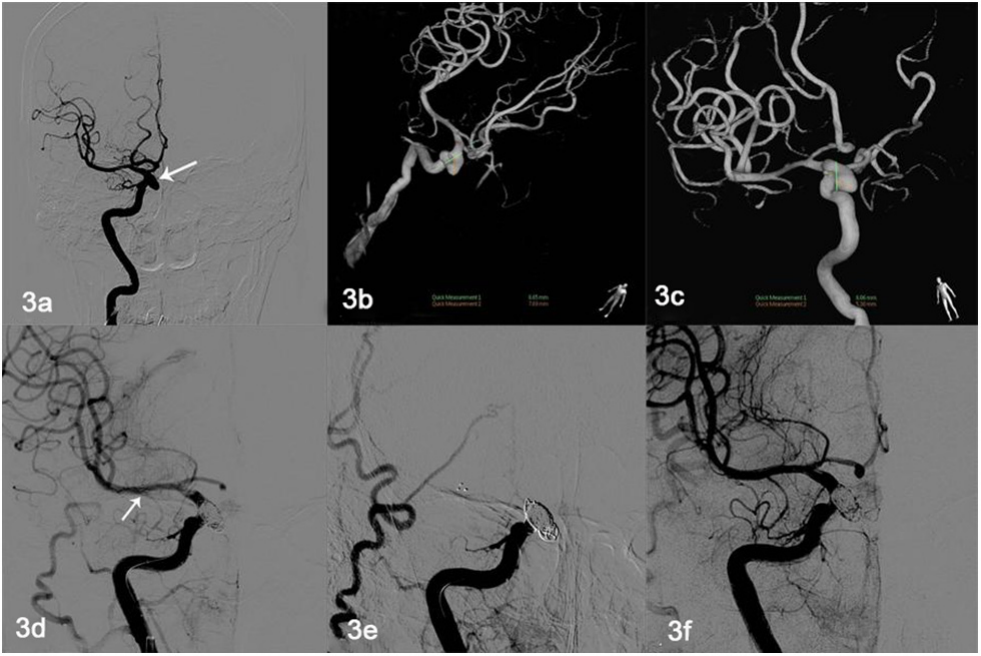
Thromboses throughout the stent in one patient. **(a–c)** Figure depicts a 46-year-old man with right carotid-ophthalmic wide-necked ruptured aneurysm (8.69 mm × 7.69 mm). (d) Solitaire AB stent (6 mm × 30 mm) assisting embolization (white arrow); there was parent artery patency. (e) Acute stent thrombosis 3 h later; and 0.3 million U urokinase was administered through the microcatheter. (f) Repeat radiography revealing expedited blood flow through the stent.

The other four cases of complications were stent thrombosis, which lasted 5 min to 8 h, and comprised two aneurysm neck thromboses and two thromboses throughout the stent. Contact thrombolysis through the microcatheter was performed immediately after the appearance of thrombosis. In the four thrombosis cases, the stents were recanalized after 0.3–0.6 million U urokinase had been injected through the catheter (Fig 3A–3F). Thrombolysis-related bleeding events did not occur in the patients who underwent contact thrombolysis.

### Follow-up

We followed 114 patients; the follow-up time was 3–37 months, and 92 patients underwent DSA examination at the 6-month follow-up. We detected 12 cases of aneurysm recurrence (12/92, 13.0%) (Tables 2 and 3), but no rupture or cerebral hemorrhage, and of which five cases (5.4%) were re-treated with intervention therapy: four with single coiling and one with Solitaire stent–assisted coiling.Among the 114 patients, 107 received good prognosis (mRS 0, 43 cases; mRS 1, 64 cases); seven had minor disability (mRS 2).

**Table 2:** Results of post-procedural occlusion and occlusion at six months.

	Post-procedure (n = 120)	Post-procedure (n = 92*)	6 Months (n = 92)
Complete occlusion	55 (45.8%)	47 (51.1%)	67 (72.8%)
Neck remnant	59 (49.2%)	40 (43.5%)	16 (17.4%)
Aneurysm remnant	6 (5.0%)	5 (5.4%)	9 (9.8%)

*28 patients were lost to follow up at six months.

**Table 3:** DSA results at six months (n = 92).

	Post-procedure	6 Months		
		No changes	Progressive occlusion	Recurrence
Complete occlusion	47	40	0	7 *
Neck remnant	40	11	26	3
Aneurysm remnant	5	2	1	2

*Two cases of neck remnant and five cases where contrast agent had entered the aneurysm intracavity.

## Discussion

Whether through clipping or intervention, intracranial wide-necked aneurysms remain a challenge to treat. Despite remodeling techniques such as the basket technique, embolization remains difficult. In 1997, Higashida and colleagues [8] were the first to report balloon-expandable coronary stents combined with coils for treating fusiform aneurysm, heralding a new era in the treatment of intracranial aneurysm.

At present, there are several stents in common use for intracranial aneurysms: the Neuroform stent (Boston Scientific/Target, Fremont, CA), the LEO stent (Balt, Montmorency, France), the SOLO stent (eV3), the Enterprise stent (Cordis Neurovascular, Miami, FL), the Solitaire AB stent (eV3) and the more recently developed flow-diverting stent (Pipeline Flex, ev3). Due to their differing designs, these stents have different characteristics, but only the Enterprise, LEO, and Solitaire AB stents are retrievable. The LEO stent is easily dislocated in half-release mode, and does not have retrievable markers that can be used to determine the critical point of withdrawal; it also has poor compliance in that it cannot expand in tortuous vascular channels. The Enterprise stent can be withdrawn once even upon 70% release, but when a short stent is inserted into a vascular channel of varying diameters, its proximal end is easily dislocated [9]. The Solitaire AB stent can be repeatedly withdrawn after being completely released as long as it is not detached, marking the advantage of the Solitaire AB stent. However, the effectiveness and safety of the Solitaire AB stent as compared with that of the Neuroform and Enterprise stents still requires validation in the form of large-sample studies [3–6].

The Solitaire AB stent is self-expandable, and its design has both the advantages of a closed-cell structure, which provides good radial support force and resistance to bending, and a tubular laser-cut design, which allows it to easily pass through tortuous vascular channels. The stent can be repeatedly released and withdrawn before being detached, therefore controlling its location and the best placement position is easy. In this study, the rate of successful stent placement was 100%, which agrees with the outcome of a multi-center prospective study by Gory et al. (98.5%) [4] and is similar to the result of other stent studies: Gentric and colleagues [10] treated 107 aneurysms using the Neuroform stent, and the successful stenting rate of their multi-center consecutive prospective study was 94.4%; Weber et al. [11] reported a prospective study in which 31 aneurysms were treated using Enterprise stents, with a successful stent placement rate of 100%.

### Efficacy of the Solitaire AB Stent

In this study, DSA immediately after embolization yielded better Raymond scores than that reported by Gory et al. [4] and Clajus et al. [6]. The former used the Solitaire AB stent to treat 64 aneurysms, and reported 42.2% complete occlusion, 39.1% neck remnant, and 18.7% aneurysm remnant. The latter reported 51% complete occlusion and 44% neck remnant. For the other stents listed earlier, the results differ between institutions. The Neuroform stent has a complete occlusion rate of 31.6–66.4% [10,12]; that for the Enterprise stent is 19.3–24.6% [11,13]. Although the different embolization techniques may influence the evaluation of the radiography results, the aneurysm complexity and surgical path conditions, and the tubular laser-cut design, closed-cell structure, and retrievability (before Guglielmi detachment) of the Solitaire AB stent allows surgeons to withdraw the stent and adjust the microcatheter if they encounter difficulties during embolization. Moreover, the Solitaire AB stent has bigger struts and less metal coverage than the Neuroform and Enterprise stents, therefore it is easier to enter and operate in the intracavity through the struts, which increases the possibility of complete occlusion.

### Complications

Stent thrombosis and hemorrhage are the most common complications of stent-assisted coil embolization of wide-necked aneurysms. In this study there were eight (6.7%, 8/120) severe complications (four each of hemorrhage and stent thrombosis). Different centers have reported markedly different complication rates, ranging from 0% to 25%, with Solitaire stent assisted embolization of wide-necked aneurysms [4,6,14–17]. Although the technical abilities of the operator is likely to be an important factor for this, so to may be the different thresholds and criteria used for determining complications. Clajus et al reviewed the data from 104 patients who were treated using the Solitaire stent. Five cases each of thrombosis and hemorrhage occurred, but the authors considered that only four procedure-related complications occurred (3.9%) [6]. Gory et al reported a retrospective study involving 64 wide-necked aneurysms, in 63 patients, that were treated using a total of 66 Solitaire stents. Eight (12.7%, 8/63) complications occurred; six thromboses and two hemorrhages, including one retroperitoneal hematoma [4]. However, in most reported series retroperitoneal hematomas and hematomas at the puncture sites in the femoral arteries are not considered to be procedure-related complications. The situation also pertains in the reporting of complications with Neuroform and Enterprise stents. The reported complication rates using these range from 0% to 50.5% [10,18–20] and 2.4% to 31.3% [19–22], respectively. Chalouhi et al used Neuroform and Enterprise stents in the embolization of 552 aneurysms. They reported a complication rate of 6.2% (31/500). This included 23 hemorrhages (6.2%), 6 thromboses (1.2%) and 2 internal carotid artery intimal tears (0.4%) [23]. Geyik et al embolized 500 wide-necked aneurysms with various kinds of stents, including Enterprise (n = 340), Solitaire (n = 98), Wingspan (n = 41), LEO (n = 16), and Neuroform (n = 5) stents. There were 21 thrombotic (4.2%) and 4 hemorrhagic (0.8%) complications [24].

In our study, the four aneurysmal hemorrhages probably resulted from mechanical irritation during deployment of the stents. And all four thrombosis complications had aneurysm rupture. Stenting of acutely ruptured aneurysms was associated with higher thromboembolic complications than when compared to stenting of unruptured aneurysms in our study. This is in accord with the findings of others [25–27]. Furthermore, patients in this cohort did not undergo platelet function testing for clopidogrel resistance, and conventional-dose anti-platelet medication may not have been sufficient for preventing stent thrombosis in patients who were probably clopidogrel-resistant.

### Mid-term Anatomic Results of Solitaire AB Stent

Ninety-two patients underwent DSA examination at the 6-month follow-up. We detected 12 cases of aneurysm recurrence (12/92, 13.0%). In our series, 28 patients were lost to the 6-month follow-up, and most of them did not have complete aneurysm occlusion (neck remnant, 19 cases; aneurysm remnant, one case). As these patients are more likely to experience recurrence compared with those who had complete occlusion, the actual recurrence rate could be similar to that of Gory et al.[15] (8/55, 14.5%).

Intracranial stents provide scaffolding for reconstructing the intimal layer of the parent artery at the aneurysm neck and may also promote progressive thrombosis [6,12,28,29]. In this study, there was progressive occlusion in 26 aneurysms (26 /92, 29.3%), which is essentially the same as that reported by Gory et al. [15] (17/55, 30.9%), and slightly lower than that reported by Clajus et al. [6] ((20/49, 39.2%). To date, the predictive factors of progressive thrombosis failure are not well known, and different stents have significantly different rates of progressive thrombosis [28,30–32]; the closed-cell design of the Enterprise and Solitaire stents may alter intra-aneurysmal hemodynamic parameters more efficiently and promote saccular thrombosis [32].

In conclusion, the Solitaire AB stent is effective with a good technical success rate and short-term effect for assisting coil embolization of wide-necked cerebral aneurysms. Its advantage is retrievability after being completely released. Nevertheless, larger sample studies and long-term follow-up are warranted to determine its long-term effects.

### Limitations

As a retrospective study, our data are prone to selection bias. There is no control group for comparing patient baseline characteristics and outcomes. In addition, the study lacks independent angiographic outcome analysis, and platelet function testing was not performed before treatment; this is a design flaw, and a clinical deficit.

## Supporting Information

S1 Fig Ethics committee (page 1). (JPG)

S2 Fig Ethics committee (page 2). (JPG)

S1 Text Ethics committee translate in English. (DOCX)
