# Supplementary figures and images for: The Use of Solitaire AB Stents in Coil Embolization of Wide-Necked Cerebral Aneurysms
Source: PLoS One. 2015 Oct 1;10(10):e0139714. doi: 10.1371/journal.pone.0139714 (PMC4591355; doi:10.1371/journal.pone.0139714)

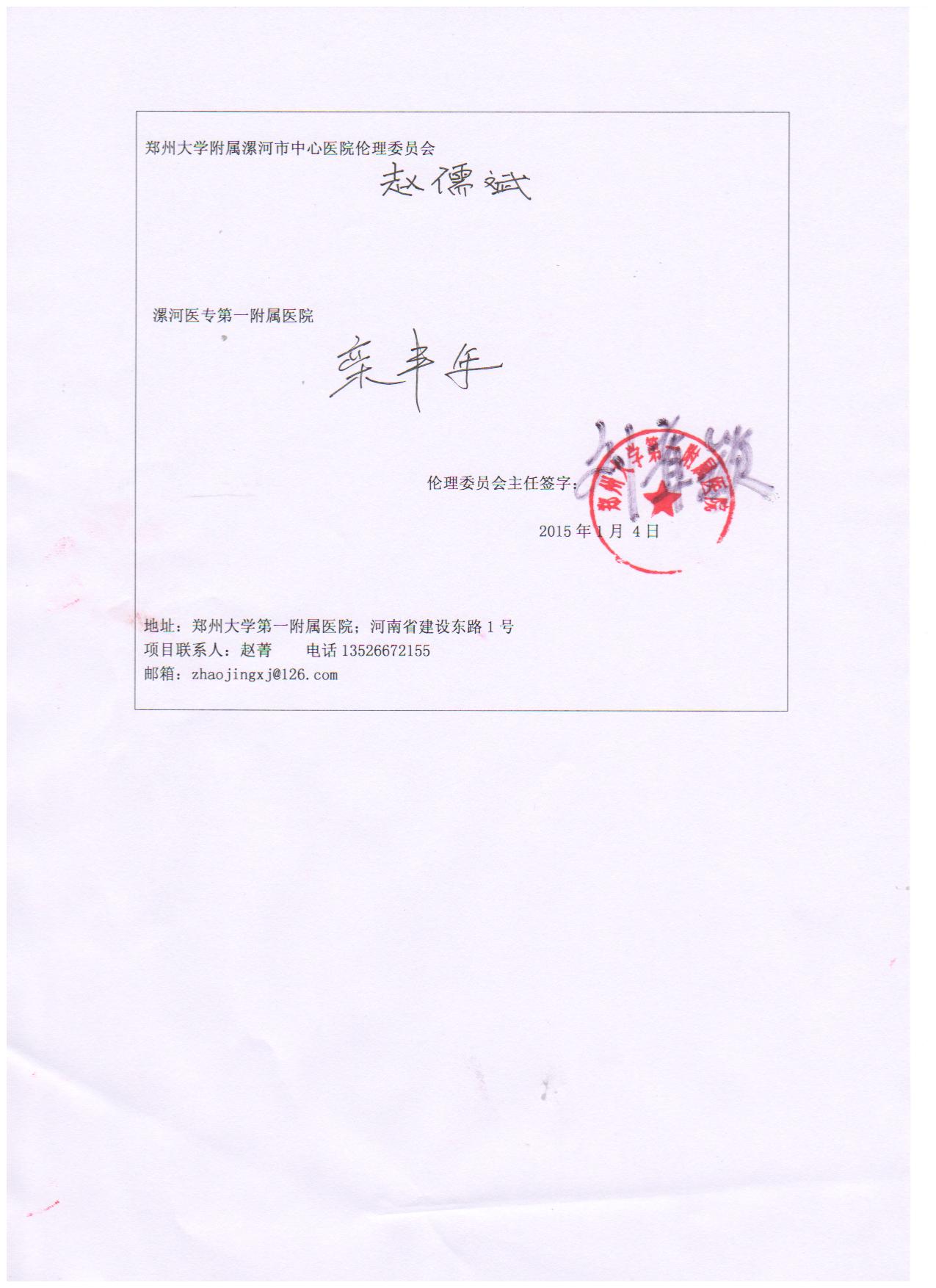

Supplement: S1 Fig — (JPG) [file pone.0139714.s001.jpg]

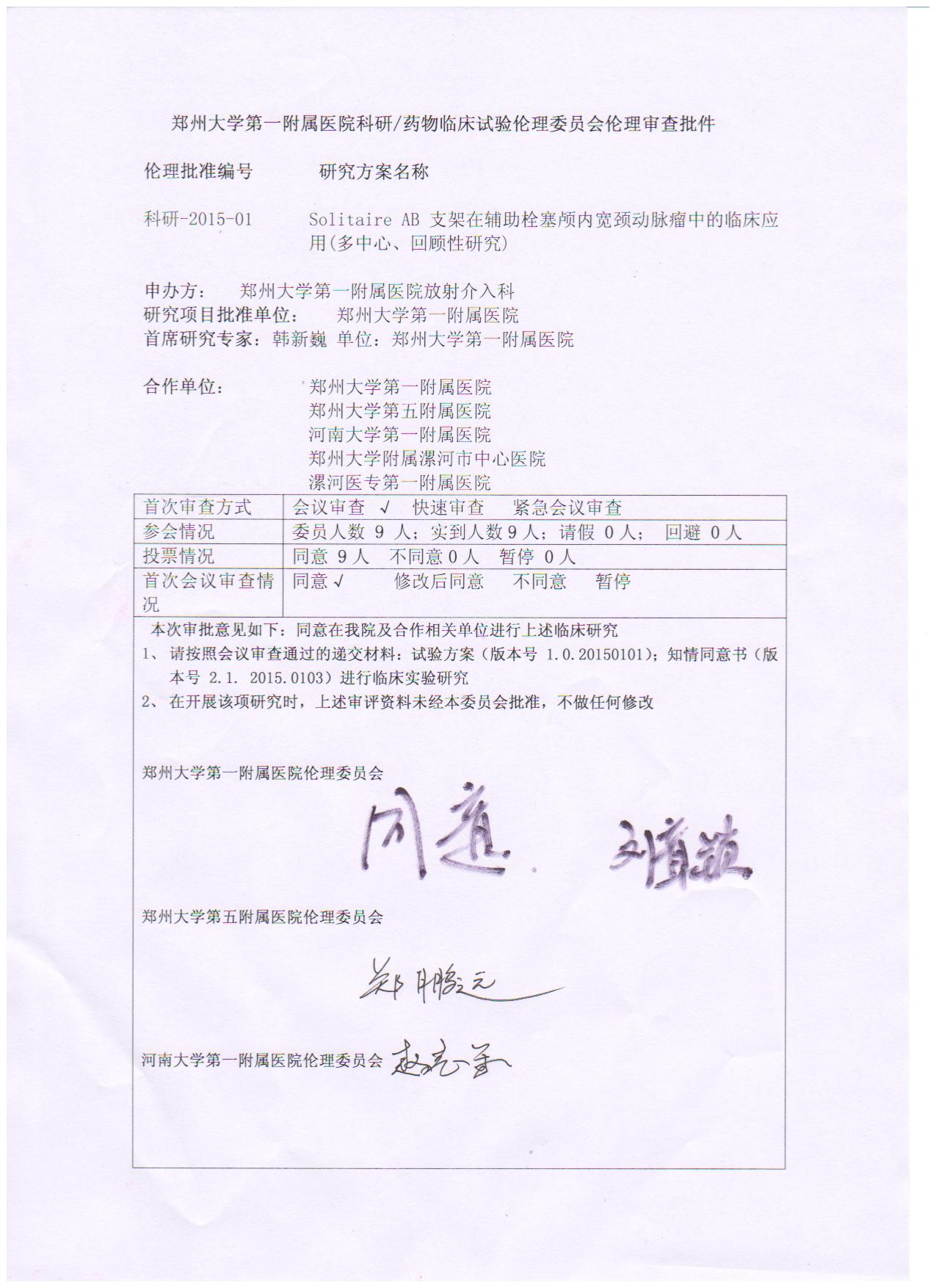

Supplement: S2 Fig — (JPG) [file pone.0139714.s002.jpg]
