# Supplementary material for: The Use of Solitaire AB Stents in Coil Embolization of Wide-Necked Cerebral Aneurysms
Source: PLoS One. 2015 Oct 1;10(10):e0139714. doi: 10.1371/journal.pone.0139714 (PMC4591355; doi:10.1371/journal.pone.0139714)
Supplement: S1 Text — (DOCX) [file pone.0139714.s003.docx]

**Ethics Statement**

**Approval number**: 科研-2015-01

**Research project**: The Use of Solitaire AB Stents in Coil Embolization of Wide-necked Cerebral Aneurysms (Multicenter, retrospective study)

**The main research person**: Xin-wei Han

**Research unit**: the First Affiliated Hospital of Zhengzhou University

the Fifth Affiliated Hospital of Zhengzhou University

the First Affiliated Hospital of Henan University

Luoyang Central Hospital of Zhengzhou University

the First Affiliated Hospital of Luohe Medical College

**The ethics committee participants**:9 persons

**Agree**: 9 persons **disagree**: 0

**Meeting decision**: Approved

the ethics committee of the First Affiliated Hospital of Zhengzhou University

**Person in charge**: Zhang-Suo Liu

the ethics committee of the Fifth Affiliated Hospital of Zhengzhou University

**Person in charge**: Peng-Yuan Zheng

the ethics committee of the First Affiliated Hospital of Henan University

**Person in charge**: Zhi-Xue Zhao

the ethics committee of Luoyang Central Hospital of Zhengzhou University

**Person in charge**: Ru-Bin Zhao

the ethics committee of the First Affiliated Hospital of Luohe Medical College

**Person in charge**: Feng-Nian Luan

**President of the ethics committee**: Zhang-Suo Liu

2015.01.04

**Address:** Jian She Road NO.1, Er Qi District, Zheng zhou City, Henan Province, China

**Moble:** +8613526672155

**Email** :zhaojingxj@126.com
